# Home treatment in acute postpartum psychosis: a case report

**DOI:** 10.1192/j.eurpsy.2025.2411

**Published:** 2025-08-26

**Authors:** J. Hidalgo-Lopez, P. Leganes, E. Serna

**Affiliations:** 1 Hospital Universitario José Germain, Leganés (Madrid); 2 Hospital Universitario Gregorio Marañón, Madrid, Spain

## Abstract

**Introduction:**

Postpartum (or puerperal) psychosis is a severe mental health condition about which there is little literature, which recommends measures to prevent separation between mother and baby in the acute crises, such as home treatment. However, there is a lack of published experiences on the subject. Here we present the case of a 36-years-old woman with this diagnosis successfully treated under a home treatment, and with history of one previous hospital admission in a conventional inpatient psychiatric care unit for the same diagnosis.

**Objectives:**

To show the specific advantages of home treatment over conventional hospitalization in acute conditions that are particularly difficult for the family environment, like the postpartum psychosis.

**Methods:**

A case report is presented alongside a qualitative analysis of the perceived experience, based on a brief semi-structured interview with the patient.

**Results:**

The patient’s own comparative experience shows less interference in the development of mother-child care and greater satisfaction under the home treatment model.

**Conclusions:**

This case helps to support home treatment as a better way of acute management of postpartum psychosis, compared to conventional hospitalization, and invites to further research on the topic.

**Disclosure of Interest:**

None Declared

